# Micropropagation and cytogenetic assessment of *Zingiber* species of Northeast India

**DOI:** 10.1007/s13205-012-0108-y

**Published:** 2012-12-30

**Authors:** Archana Das, Vigya Kesari, Latha Rangan

**Affiliations:** Department of Biotechnology, Indian Institute of Technology, Guwahati, 781 039 Assam India

**Keywords:** Axillary bud, Micropropagation, Northeast India, RAPD, *Zingiber moran*

## Abstract

An improved micropropagation protocol was developed for *Zingiber moran* and *Z. zerumbet*, two wild species of the genus *Zingiber*, found in Northeast India. The effects of growth regulators, sugar concentrations, and nutrients were tested on the rate of shoot initiation and multiplication. An increase in proliferation and multiplication occurred in modified Murashige and Skoog (MS) medium supplemented with benzyladenine and kinetin. About 2 % sucrose and 0.7 % agar were found to be the optimum for shoot multiplication and regeneration. Naphthalene acetic acid at 0.5 mg/L produced the best rooting response for both the species. Regenerated plantlets were acclimatized successfully and cytogenetic stability was confirmed by RAPD profiling and ploidy checks.

## Introduction

The genus *Zingiber* (family Zingiberaceae) is a perennial herb which posses underground tuberous aromatic rhizomes and currently comprises of approximately 85 species and has a widespread occurrence in East Asia and tropical Australia (Mabberley 1990). *Zingiber moran* and *Z. zerumbet* are two wild species invariably found in Northeast (NE) India. *Z. moran* is an endemic species restricted to certain locations in NE India and shares morphological similarity with *Z. officinale*, the common ginger. The *Z. moran* rhizome is much smaller in size, is dirty white in color with a characteristic strong aroma and has immense medicinal value, being regularly used in home-made therapies by local folk. It is popularly known as “Moran aada” in the local language of Assam and is used as an excellent expectorant, carminative, diuretic, stimulant, and in many other households for therapeutic measures. The *Z. zerumbet*, commonly known as ‘Shampoo ginger’ has been traditionally used for the treatment of fever, constipation and to relieve pain (Tushar et al. 2010). It also possesses antipyretic, analgesic (Somchit et al. 2005), anti-inflammatory properties, and chemo-preventive activities (Nakamura et al. 2004). In spite of these medicinal properties, little work has been done on cultivation or conservation of these plants at the rural level and the species are facing the danger of extinction. In fact, *Z. moran* has already been categorized as a rare species. The most appropriate strategy for conserving endangered species is to protect them in their natural habitat. However, with an increase in the rate of deforestation, many species became extinct even before such investigations can be carried out that could lead to cultivation and conservation. In vitro propagation is a true-to-type multiplication technique, which provides uniform plants with genetic identity (Das et al. 2010). This technique has been applied for the conservation of more than 170 endangered plant species derived from 60 different families (Pence 1999).

Tissue culture techniques have been applied typically when traditional methods of propagation have either failed or proved inadequate. Until date, there has been no any report on the in vitro propagation of *Z. moran*. In contrast, there are a few reports on the micropropagation of *Z. zerumbet* from different countries (Hsu et al. 1991; Nalawaade et al. 2003; Chan and Thong 2004; Stanly and Keng 2007), but none is from India. The present study is an effort to optimize the in vitro protocols for direct plantlet regeneration, rapid multiplication, and effective conservation of the two important wild medicinal plants as well as to establish genetic purity and cytogenetic stability of the in vitro raised plants by RAPD profiling and ploidy check, respectively. The results derived from these studies will contribute to further research and conservation of endemic species of the family Zingiberaceae.

## Materials and methods

### Plant material and explant preparation

Mature rhizomes of *Z. moran* and *Z. zerumbet* were collected during rainy season from Kamrup, Barpeta, Nagaland, and Sivasagar. The rhizomes were placed in sand and soil mixture (1:3) in a pot and kept for sprouting. After 8–10 days, axillary buds that sprouted from the rhizomes were used as explants. The buds were thoroughly washed under running tap water for 30 min to remove dust and soil particles. They were then treated with 0.1–0.2 % Bavistin solution (BASF, India), a systemic fungicide for 20 min under continuous agitation followed by rinsing with sterile distilled water. Buds were subsequently surface sterilized with 0.1 % Na-hypochlorite solution along with (2 % v/v) Tween 20 for 5 min, rinsed with sterile water three times and treated with 70 % ethanol for 2 min. A subsequent second surface sterilization treatment included exposure to 0.1 % (m/v) mercuric chloride for 10 min followed by five rinses with sterile water.

### Inoculation and establishment of explants

Sterilized buds were excised from both ends and inoculated into culture tubes (Borosil, Mumbai, India) containing the standard MS medium (Murashige and Skoog 1962) and modified MS medium (MSR) fortified with the following supplements—yeast extract (300 mg/L), casein hydrolysate (100 mg/L) with 0.8 % agar (Himedia, Seward Medical, London, UK) and 3 % sucrose. The pH of the media was adjusted to 5.8 and the tubes were autoclaved at 121 °C for 15 min. The cultures were incubated at 25 ± 2 °C, with a 16 h photoperiod and irradiance of 40 μmol m^-2^/s provided by cool white fluorescent tubes. MS medium supplemented with different concentrations of benzyladenine (BA) and kinetin (Kn) were tried individually and in combination for shoot multiplication and maintenance.

### Media and culture conditions

Basal MS medium and modified MSR medium supplemented with different combinations and concentrations of plant growth regulators (PGR) and varying sucrose concentrations viz; 10 g, 20 g and 30 g/L was used in the study (Tables 1, 2). Standard procedures were followed for media preparation and maintenance (Vincent et al. 1992). Multiplication medium fortified with various concentrations of agar (6.0, 7.0, and 8.0 g/L) was used to demonstrate the most effective level of the gelling agent. Subculturing was carried out after 3 and 8 weeks of culture. After 6 weeks, the number of explants responded, shoots per explant, shoots length were recorded whereas the rooting percentage, number of roots per shoot and root length were recorded after 10 weeks of culture.

**Table 1:** Effect of medium and growth regulators on percentage response, number of shoots and length of the longest shoot in *Z. moran* (after 6 weeks of culture)

Growth Regulators	Concentration (mg/L)	MS medium response (%)	Mean number of shoots/explant	Mean length of the longest shoot (cm)	MSR medium response (%)	Mean number of shoots/explant	Mean Length of the longest shoot (cm)
BA	1.0	NR	–	NR	36.66 ± 0.57	2.55 ± 0.05ef	1.62 ± 0.06a
BA	2.0	46.66 ± 0.57	3.69 ± 0.07a	2.50 ± 0.04ab	73.33 ± 0.57	3.70 ± 0.09ac	2.70 ± 0.03bd
BA	3.0	33.33 ± 0.57	1.80 ± 0.04ab	2.00 ± 0.05ab	56.66 ± 0.57	1.84 ± 0.06bcd	2.02 ± 0.02f
Kn	1.0	NR	–	–	56.66 ± 0.57	1.81 ± 0.06af	1.70 ± 0.02ae
Kn	2.0	46.60 ± 0.57	2.21 ± 0.06bc	2.6 ± 0.03ce	76.66 ± 1.15	2.80 ± 0.07ab	2.94 ± 0.03c
Kn	3.0	36.60 ± 1.15	2.17 ± 0.4c	1.84 ± 0.03ae	60.0 ± 1.0	1.52 ± 0.05cd	2.30 ± 0.03d
BA + Kn	1.0 + 1.0	63.33 ± 0.57	4.77 ± 0.09ab	4.22 ± 0.04cd	86.66 ± 0.57	7.42 ± 0.13cf	4.92 ± 0.06fg
BA + Kn	2.0 + 1.0	53.33 ± 0.57	2.44 ± 0.06cd	2.97 ± 0.04ab	60.0 ± 1.0	3.00 ± 0.08ab	3.30 ± 0.03g
BA + Kn	3.0 + 1.0	50.0 ± 1.0	2.56 ± 0.05ab	2.53 ± 0.03ae	63.33 ± 0.57	3.18 ± 0.07c	3.12 ± 0.04bd
BA + Kn	1.0 + 2.0	56.66 ± 0.57	3.00 ± 0.07a	1.90 ± 0.02af	60.0 ± 1.0	2.45 ± 0.06f	2.10 ± 0.05d
BA + Kn	2.0 + 2.0	83.33 ± 0.57	6.17 ± 0.12bcd	4.98 ± 0.05de	100 ± 0	9.16 ± 0.17de	5.81 ± 0.06bc
BA + Kn	3.0 + 2.0	53.30 ± 0.57	3.06 ± 0.08ce	2.43 ± 0.03ef	56.66 ± 0.57	3.8 ± 0.07d	2.50 ± 0.02ae
BA + Kn	1.0 + 3.0	56.66 ± 0.57	3.00 ± 0.09	1.93 ± 0.03be	76.66 ± 0.57	2.71 ± 0.06fg	2.19 ± 0.03f
BA + Kn	2.0 + 3.0	53.33 ± 0.57	2.42 ± 0.06	1.92 ± 0.03eg	60.0 ± 1.0	2.55 ± 0.06f	2.01 ± 0.05g
BA + Kn	3.0 + 3.0	NR	–	–	33.33 ± 0.57	1.2 ± 0.05e	1.50 ± 0.05g

Mean ± SE, *n* = 10. Means followed by the same letters in each column are not significantly different at *P* < 0.05 (Duncan’s multiple range test)

**Table 2:** Effect of medium and growth regulators on percentage response, number of shoots and length of the longest shoot in *Z. zerumbet* (after 6 weeks of culture)

Growth Regulators	Conc. (mg/L)	MS medium response (%)	Mean no of shoots/explant	Mean shoot length (cm)	MSR medium response (%)	Mean no of shoots/explant	Mean shoot length (cm)
BA	1.0	3.33 ± 0.57	3.00 ± 0.06a	1.39 ± 0.03ab	26.66 ± 0.57	2.37 ± 0.05bc	1.58 ± 0.03d
BA	2.0	46.66 ± 1.52	4.00 ± 0.08ac	1.81 ± 0.02b	70.00 ± 1.0	3.42 ± 0.06e	1.90 ± 0.03ab
BA	3.0	40.00 ± 1.0	2.75 ± 0.06abc	1.45 ± 0.03b	63.33 ± 0.57	2.42 ± 0.08ac	1.94 ± 0.04a
Kn	1.0	13.30 ± 0.63	2.50 ± 0.05bc	1.75 ± 0.03cd	43.33 ± 0.57	2.30 ± 0.06ab	1.44 ± 0.03abc
Kn	2.0	40.00 ± 1.0	2.66 ± 0.07c	1.8 ± 0.05de	86.66 ± 0.57	2.50 ± 0.05a	1.41 ± 0.05def
Kn	3.0	40.00 ± 1.0	1.58 ± 0.05b	1.75 ± 0.04d	43.33 ± 0.57	2.58 ± 0.06b	2.21 ± 0.04d
BA + Kn	1.0 + 1.0	86.66 ± 1.52	4.50 ± 0.10ab	1.88 ± 0.09a	76.66 ± 0.57	4.17 ± 0.12f	1.86 ± 0.05de
BA + Kn	2.0 + 1.0	60.00 ± 1.0	3.38 ± 0.06c	4.40 ± 0.07f	60.00 ± 1.0	3.27 ± 0.10f	4.36 ± 0.10cd
BA + Kn	3.0 + 1.0	56.66 ± 0.57	3.05 ± 0.07ad	1.92 ± 0.08ae	66.66 ± 0.57	3.60 ± 0.09ef	3.98 ± 0.09ab
BA + Kn	1.0 + 2.0	63.33 ± 0.57	3.26 ± 0.08cd	2.18 ± 0.04ef	53.33 ± 0.57	3.31 ± 0.10ae	2.93 ± 0.03bc
BA + Kn	2.0 + 2.0	96.66 ± 0.57	5.65 ± 0.08cbd	3.60 ± 0.10e	100.00 ± 0.0	7.23 ± 0.14bc	2.88 ± 0.04b
BA + Kn	3.0 + 2.0	70.00 ± 2.64	3.47 ± 0.10cb	3.09 ± 0.09c	6.00 ± 1.0	5.50 ± 0.25cd	3.43 ± 0.07d
BA + Kn	1.0 + 3.0	56.66 ± 0.57	2.70 ± 0.08c	2.15 ± 0.04d	63.33 ± 1.52	6.78 ± 0.15d	3.91 ± 0.05e
BA + Kn	2.0 + 3.0	43.33 ± 0.57	2.53 ± 0.07ab	2.1 ± 0.03bc	46.66 ± 0.57	2.42 ± 0.06d	2.36 ± 0.04f
BA + Kn	3.0 + 3.0	NR	–	–	NR	–	–

Mean ± SE, *n* = 10. Means followed by the same letters in each column are not significantly different at *P* < 0.05 (Duncan’s multiple range test)

### Acclimatization of plantlets

Healthy and rooted shoot clumps were removed from culture tubes and washed thoroughly with tap water and subsequently planted in poly-bags containing sand and clay at the ratio of 1:4. Plantlets were maintained in a greenhouse under semi-shade and high humidity (RH 80 %) with a 16 h photoperiod at 28 ± 2 °C for hardening. Intermittent mist was supplied for 30 s at 15 min intervals. Percentage survival was determined after 60 days. Later, the plantlets were isolated carefully and transferred to the garden for further establishment.

### Statistical analysis

Each treatment contained 10 replicates and was repeated twice. All experiments were conducted in a completely randomized manner. After 6 weeks of culture period, the percentage of explants initiating shoot buds, mean number of multiple shoot buds and mean length of the longest shoot per explant were recorded. For rooting, percentage rooting, mean root number, and mean root length per explant were measured after 10 weeks of culture. The data were analyzed using one-way ANOVA (SPSS 16.0 version, 2008) and significant differences between treatment mean were assessed using Duncan’s multiple range test (DMRT) at a 5 % probability level (*P* < 0.05).

### Cytogenetic stability of regenerated plants (RAPD analysis and ploidy check)

For RAPD analysis, 10 regenerated plants of *Z. moran* and *Z. zerumbet* collected from 60-day-old plantlets were randomly picked. Total genomic DNA of the mother plant and tissue culture raised progenies were extracted from fresh tender leaves using a SDS extraction protocol (Kesari et al. 2009; Das et al. 2010). The quality and quantity of the extracted DNA was confirmed to be consistent both spectrophotometrically and by running on 1.0 % agarose gels containing 0.5 μg/ml of EtBr.

PCR amplification of the genomic DNA was carried out using 10 arbitrary decamer oligonucleotide primers (Operon Technologies, Almeda, USA). Each reaction mixture of 0.02 ml contained 50 μg/ml of template DNA, 1× assay buffer (100 mM Tris sulfonic acid, pH 8.8, 15 mM MgCl_2_, 500 mM KCl and 0.1 % gelatin), 0.2 mM each dNTPs (*Banglore Genei*, Bangalore, India), 5 pmol of each primer and 0.5 U of *Taq* polymerase (Banglore Genei). PCR amplification was carried out in a mini thermal cycler (Applied Biosystems, Foster City, CA, USA) programmed for 40 cycles. The initial denaturation step of 5 min at 94 °C, was followed by 40 cycles of 45 s at 94 °C, annealing for 1 min at 32 °C, and extension at 72 °C for 2 min and a final extension cycle of 10 min at 72 °C. The amplification products were electrophoresed in 1.3 % agarose gels in 1× TAE buffer (50× stock solution contained 2 M Tris, 0.5 M EDTA and glacial acetic acid). The gels were visualized and photographed under UV radiation by a gel documentation system (BioRad, Hercules, USA). The size of the amplification products was estimated using a λ DNA marker (Banglore Genei). To avoid any ambiguity, only the bands with higher intensity were considered.

Further, ploidy level of the cloned plants was checked by chromosome counting after acclimatization. Root tips of the mother plant and regenerated plants in both the species were fixed during somatic cell division in the morning hours (9.00–10.30 am). Fixed root tips were acid hydrolyzed and stained with Acetocarmine (2 %) for 2 h. Slides were prepared and the metaphase chromosomes were counted for each species under study. Regenerated plants were studied individually to see if there was any variation.

## Results

### Culture initiation and multiplication

High rate of contamination was a hurdle in establishment of the aseptic cultures in *Zingiber* species which was overcome by twofold surface sterilization with 0.1 % Na-hypochlorite solution along with (2 % m/v) Tween 20 for 5 min and mercuric chloride 0.1 % (m/v) for 10 min before inoculation.

Four to 6 weeks after inoculation, new shoots emerged from the rhizome buds. Various combinations of PGRs were used with MS and MSR media for culture initiation. The explants on different combinations and concentrations of PGRs showed a large variability in culture response. *Z. zerumbet* showed response to 14 PGR combinations tried, where as *Z. moran* responded to all the 15 combinations used in the study. However, MS medium with BA (1 mg/L) showed poor response in *Z. zerumbet* and no response at all in case of *Z. moran.* In addition, the PGR combination of BA (3 mg/L) along with Kn (3 mg/L) showed no response to *Z. zerumbet*. The greatest response for enhanced induction of rhizome buds in *Z. zerumbet* were recorded in MS (96.66 %) and MSR medium (100 %) supplemented with 2 mg/L BA + 2 mg/L Kn, respectively, after 2 weeks; where as that of *Z. moran* was found to be 83.33 % (MS) and 100 % (MSR) with the same combinations of PGR used.

In vitro raised explants responded to induced multiple shoots in different degrees in two different media tried viz., MS and MSR media with varying concentrations and combinations of cytokinins and auxins. The MSR medium containing BA (2 mg/L) + Kn (2 mg/L) proved to be best medium for shoot proliferation in case of both the *Zingiber* species studied. Rhizome buds started to proliferate soon after 10 days of inoculation in MSR medium compared to the same combination tried in MS medium, which responded after about 2 weeks. Though shoot multiplication was observed in single treatment of cytokinins, the rate of multiplication was poor. Among different range of cytokinins tested, BA (2 mg/L) offered better provision to develop an average of 4 and 3.7 shoots per explant in *Z. zerumbet* and *Z. moran*, respectively. The presence of BA along with Kn in the medium markedly increased the number of shoots produced per explant. The highest shoot induction was found in 2 mg/L BA + 2 mg/L Kn, which produced highest number of shoots in both the species studied (9 in *Z. zerumbet* and 12 in *Z. moran*). Higher concentration of BA was found to be inhibitory in shoot multiplication irrespective of the Kn concentrations used.

The highest mean number of buds per explants was also recorded in the same hormone combination which was 6.2 in MS and 9.2 in MSR medium for *Z. moran*. MSR medium along with BA and Kn with concentrations (2 mg/L + 2 mg/L) showed the highest regeneration as well as multiplication frequency followed by BA (1 mg/L) + Kn (1 mg/L) in both the *Zingiber* species (Tables 1, 2). Beneficial effect of Kn when treated along with BA attributed to the improvement of multiplication in the present study. The application of BA at 2 mg/L seemed to be suitable for optimum multiplication as concentrations above and below this level reduced shoot production. The highest mean length of the longest shoot in the two medium was also recorded as 4.98 cm in MS and 5.56 cm in MSR supplemented with BA and Kn 2 mg/L each for *Z. moran.* Whereas for *Z. zerumbet*, a highest mean shoot length of 3.6 cm was obtained in MS medium with the same PGR combinations. However, in MSR medium with BA at 2 mg/L and Kn at 1 mg/L gave the best shoot length of 4.36 cm of all (Table 2).

### Effect of agar and carbon source

In case of both the *Zingiber* species, the agar at a concentration of 0.7 % was found to be optimum for both regeneration and multiplication. Sucrose concentration at 2 % showed greater number of multiple shoots when used with MSR medium compared to that used at same concentration of sucrose with MS medium. However, 3 % sucrose with MS medium has been used invariably by many workers for members of Zingiberaceae with better results (Tyagi et al. 2004; Stanly and Keng 2007).

### Rooting response and acclimatization

**Fig. 1:**
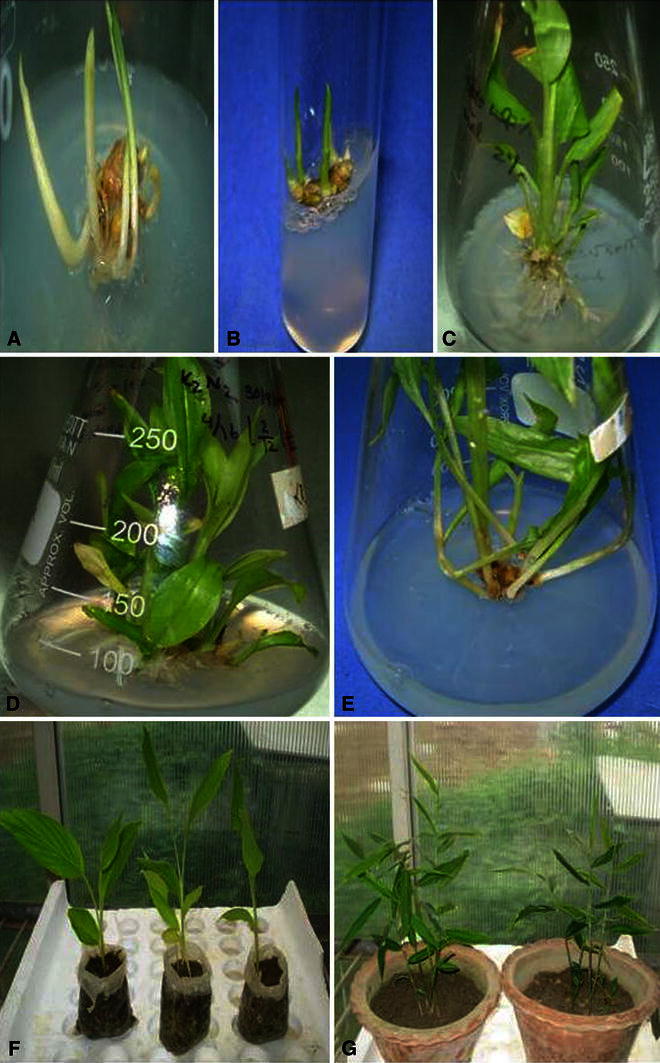
Plant regeneration in *Zingiber* species: multiple shoots sprouting from axillary buds of *Z. zerumbet* (**a**), *Z. moran* (**b**) in MSR medium supplemented with BA and Kn after 4 weeks of culture period; Profuse shoots with well developed roots in *Z. zerumbet* (**c**); Multiple shoots in *Z. zerumbet* (**d**) and in *Z. moran* (**e**) in MSR medium after 6 weeks of culture; 2 months old hardened plants of *Z. zerumbet* (**f**) and *Z. moran* (**g**)

The regenerated plants were subcultured in rooting media after 6–8 weeks of culture. Half strength of both MS and MSR media along with three different auxins were studied for rooting response of *Z. zerumbet* and *Z. moran* in vitro. All the three auxins IAA, IBA and NAA (0.5 and 1.0 mg/L each) revealed higher rooting ability at lower concentrations. However, the higher concentrations used also produced roots at a low rate and lengths of the roots were also shorter. NAA at 0.5 mg/L showed the highest about 90 and 86.6 % rooting in *Z. moran* and *Z. zerumbet*, respectively (Table 3). This was followed by IBA (0.5 mg/L) for *Z. moran* (73.3 %) and IAA (0.5 mg/L) for *Z. zerumbet* (66.6 %), respectively. Actively growing plantlets with profuse root system were transferred to greenhouse after 30–60 days of culture and acclimatized. About 90 % shoots survived in non-sterile potting mixture. Hardened plantlets were found to grow healthily with 80 % (*Z. moran*) and 100 % (*Z. zerumbet*) survival in after 4 weeks of repotting. After 35–45 days of transfer, new leaves were also developed in the in vitro shoots (Fig. 1f, g).

**Table 3:** Effect of auxin treatments on in vitro rooting in shoots of *Z. moran* and *Z. zerumbet* cultured in half-strength MS medium (after 10 weeks of culture)

Growth regulators	Concentration (mg/L)	*Z. moran response* (%)	Root number/shoot	Root length (cm)	*Z. zerumbet response* (%)	Root number/shoot	Root length (cm)
NAA	0.5	90.0 ± 1.0	5.21 ± 0.08a	5.41 ± 0.09ab	86.6 ± 0.57	7.12 ± 0.12ab	5.51 ± 0.70ab
NAA	1.0	56.6 ± 1.15	1.88 ± 0.06ab	2.05 ± 0.05b	60.0 ± 0.0	3.05 ± 0.06b	3.55 ± 0.08a
IAA	0.5	66.6 ± 0.57	3.77 ± 0.06b	3.45 ± 0.05c	66.6 ± 0.57	4.14 ± 0.12c	3.8 ± 0.11a
IAA	1.0	50.0 ± 1.0	1.73 ± 0.07c	2.11 ± 0.02a	43.3 ± 0.57	2.0 ± 0.05c	2.02 ± 0.04c
IBA	0.5	73.3 ± 0.57	3.59 ± 0.07d	3.25 ± 0.05c	56.6 ± 1.15	3.94 ± 0.08a	3.13 ± 0.05d
IBA	1.0	53.3 ± 1.15	1.82 ± 0.05a	2.11 ± 0.02a	36.6 ± 0.57	2.07 ± 0.04d	1.9 ± 0.04d

Mean ± SE, *n* = 10. Means followed by the same letters in each column are not significantly different at *P* < 0.05 (Duncan’s multiple range test)

### Cytogenetic assessment

**Fig. 2:**
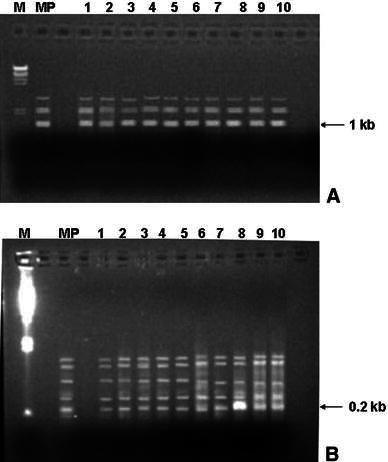
RAPD profiles of micropropagated plants of *Zingiber* species using the decamer primers: *Z. zerumbet* with primer OPA 06 (**a**), *Z. Moran* with primer OPA 03 (**b**). *Lane* M-DNA marker, *laneMP* DNA from mother plant, *lanes* 1–10 DNA from micropropagated plants

**Fig. 3:**
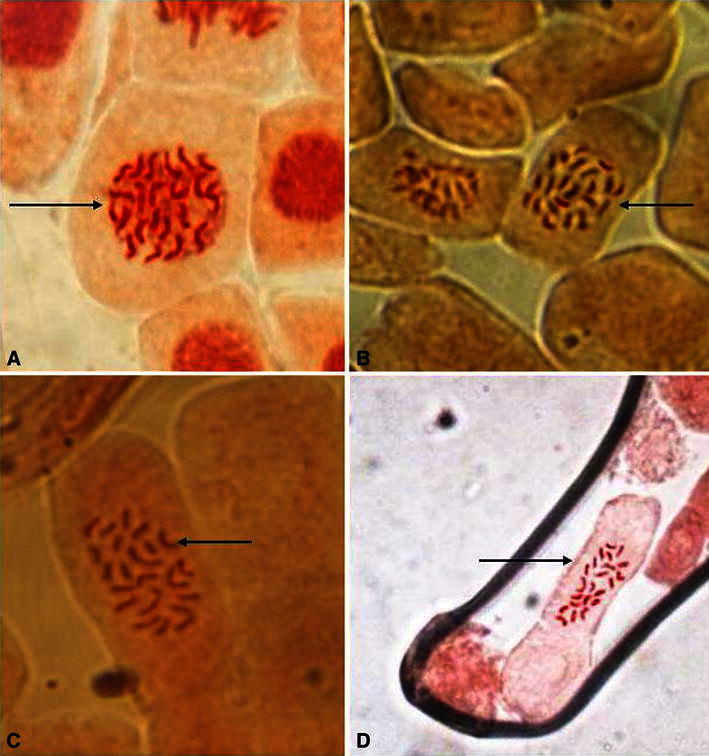
Metaphase stages showing chromosome numbers of *Z. moran* mother plant (**a**), regenerated plant (**b**); *Z. zerumbet* mother plant (**c**) and regenerated plant (**d**)

Out of the 10 different RAPD primers tested, 8 and 6 primers produced clear and scorable bands in *Z. moran* and *Z. zerumbet*, respectively, in this study. For *Z. moran*, 45 scorable bands were produced by 8 RAPD primers ranging from 500 to 3,000 bp in size. Similarly, for *Z. zerumbet*, six selected RAPD primers generated 36 reproducible bands of 200–1800 bp in size. The amplification products were monomorphic across all the micropropagated plants along with the mother plant, which confirmed the genetic fidelity of these plantlets. Representative RAPD profiles produced for the two species are depicted in Fig. 2a, b. Moreover, the ploidy level of the regenerated plants were checked by chromosome study and counting the number of chromosomes and compared with that of the mother plants. The number of chromosome was found 2*n* = 22 in *Z. moran* (Fig. 3a, b) and *Z. zerumbet* (Fig. 3c, d), respectively, in both donor and in vitro raised plants. The stable nature of the regenerants was thus ascertained by consistency of chromosome numbers.

## Discussion

Methods of culture initiation and multiple shoot regeneration are well established in *Z. officinale* (Khatun et al. 2003; Sultana et al. 2009) and similar protocols have been effectively used for *Curcuma* species (Tyagi et al. 2004; Das et al. 2010). The effect of different growth regulators and culture conditions on in vitro multiplication and rhizome formation was studied by Rout et al. (2001) for *Z. officinale*.

Contamination is a major problem in rhizomatous plants during initiation and successful establishment of aseptic cultures (Borthakur et al. 1999). In *Zingiber* species, responding percentage and the contamination rate in in vitro studies was highly dependent on the time of collection. Rainy season, being the most favorable time for initiation of culture when the buds are in actively growing state adventitious shoots developed from 80 % of the explants and rate of contamination was also less. In vitro seasonal effect on bud growth has been reported in *Z. zerumbet* and *Curcuma zedoaria* (Stanly and Keng 2007) and *Curguligo orchioides* (Wala and Jasrai 2003).

In the present investigation, multiplication was found to occur by development of axillary buds, which is ideal for maintaining genetic stability. However, the rate of bud multiplication was significantly different according to the various concentrations and combination of growth regulators used. Although explants showed a fair response to individual cytokinins used, the combinations of two regular cytokinins (BA and Kn) were found to be ideal for shoot multiplication (Table 2). Similar results were found by Anish et al. (2008) in *Bosenbergia pulcherrima*, a threatened ginger. However, persistence of explants in culture media containing higher concentration of cytokinins suppressed shoot elongation in present study which is contrary to what has been reported by other researchers who used rather high concentrations of plant growth regulators for the multiple shoot formation for some of the *Zingiberaceae* species (Khatun et al. 2003; Chan and Thong 2004; Bharalee et al. 2005; Sultana et al. 2009). Prathanturarug et al. (2004) reported that MS medium supplemented with 3 % (w/v) sucrose, BA 35.5 μM and 0.5 μM NAA induced the formation of 6.1 shoots per explants in *Z. petioletum*. Bharalee et al. (2005) found that MS medium supplemented with 4 mg/L BA and 1.5 mg/L NAA was the best medium for shoot multiplication for the genus *Curcuma.* Similarly, shoot induction rate was much slower in *Z. moran* and *Z. zerumbet* than other species of Zingiberaceae. Our results indicated that 1 mg/L BA and 1 mg/L Kn in the MSR medium was sufficient for the induction of multiple shoots from the buds and shoots of *Z. moran* and *Z. zerumbet.* The synergistic effect of cytokinins (BA and Kn) has been found to be enhancing in shoot initiation and multiplication. Micropropagation of *Z. zerumbet* has been standardized using the shoot tip explants earlier by Hsu et al. (1991) who found 4.78 shoots per responding explants with MS basal medium supplemented with 4 mg/L of BA. Our results show a better result in contrast where BA in combination with Kn (2 mg/L each) revealed 9 and 12 shoots per explant in case of *Z. zerumbet* and *Z. moran*, respectively.

Sucrose is widely used as a standard carbon source for plant tissue culture, and different concentrations and different osmotic environments have been used (Das et al. 2010). Shoot length and number significantly increased when sucrose was added to medium at a lower percentage. Sucrose 2 % were found to be most suitable for shoot multiplication for both species. Concentrations higher than 3 % caused a defoliating effect on the *Zingiber* plantlets. Similar observation was reported by Barthakur and Bordoloi (1992) in *Curcuma* species. However, higher concentration of sugar source has been found to be ideal for in vitro micro rhizome production in *Z. officinale* (Zheng et al. 2008).

Assessing the genetic purity of in vitro raised plants using RAPD has been proved to be an efficient tool for many plant species (Rout and Das 2002; Hussain et al. 2008). The source of the explants and mode of regeneration (somatic embryogenesis/organogenesis/axillary bud multiplication) are known to play a major role in determining the presence or absence of variation. Use of rhizomatous buds as explants for micropropagation lowers the risk of genetic instability as the organized meristem is generally more resistant to genetic changes that might occur by indirect regeneration (Salvi et al. 2002). Our results coincide with findings of Suri et al. (1999) who found better genetic stability in regenerated plantlets obtained from rhizomes compared to leaf explants. Moreover, the cytogenetic stability of the regenerated plants was also checked by studying the chromosome numbers and comparing those with the mother plant. Ploidy status of in vitro grown regenerants of *Curcuma longa* was reported by Panda et al. (2007). Polyploidy is common among the members of Zingiberaceae. However, no reports are known on cytogenetic stability testing of micropropagated plants in *Zingiber*. It was a strong attribute to confirm that the tissue culture raised plantlets did not develop any difference in chromosome number in spite of long in vitro conditions. Hence, the plants from both the species were exactly the clones of the donor plant.

The present study describes an efficient protocol for micropropagation of *Z. moran* and *Z. zerumbet* from NE India. As it produces shoots in absence of an intermediate callus phase, it can be used as an efficient method for clonal multiplication, source of disease free planting material and conservation of these wild, valuable, and endemic species. The findings would definitely be useful for future study of the endemic *Z. moran* or other Zingiberaceae species which are yet to be studied from NE region of India.

## Abbreviations

bp: Base pairs
MS: Murashige and Skoog
RAPD: Random amplified polymorphic DNA
